# Longitudinal lung cancer prediction convolutional neural network model improves the classification of indeterminate pulmonary nodules

**DOI:** 10.1038/s41598-023-33098-y

**Published:** 2023-04-15

**Authors:** Rafael Paez, Michael N. Kammer, Aneri Balar, Dhairya A. Lakhani, Michael Knight, Dianna Rowe, David Xiao, Brent E. Heideman, Sanja L. Antic, Heidi Chen, Sheau-Chiann Chen, Tobias Peikert, Kim L. Sandler, Bennett A. Landman, Stephen A. Deppen, Eric L. Grogan, Fabien Maldonado

**Affiliations:** 1 Department of Medicine, Division of Allergy, Pulmonary and Critical Care Medicine, Vanderbilt University Medical Center, Nashville, TN USA; 2 Department of Radiology, West Virginia University, Morgantown, WV USA; 3 Department of Surgery, Vanderbilt University Medical Center, Nashville, TN USA; 4 Department of Biostatistics, Vanderbilt University Medical Center, Nashville, TN USA; 5 Department of Medicine, Division of Pulmonary and Critical Care Medicine, Mayo Clinic, Rochester, MN USA; 6 Department of Radiology and Radiological Sciences, Vanderbilt University Medical Center, Nashville, TN USA; 7 Department of Engineering and Computer, Vanderbilt University, Nashville, TN USA; 8 Department of Thoracic Surgery, Vanderbilt University Medical Center, Nashville, TN USA

**Keywords:** Predictive markers, Lung cancer, Cancer imaging, Biomarkers, Oncology

## Abstract

A deep learning model (LCP CNN) for the stratification of indeterminate pulmonary nodules (IPNs) demonstrated better discrimination than commonly used clinical prediction models. However, the LCP CNN score is based on a single timepoint that ignores longitudinal information when prior imaging studies are available. Clinically, IPNs are often followed over time and temporal trends in nodule size or morphology inform management. In this study we investigated whether the change in LCP CNN scores over time was different between benign and malignant nodules. This study used a prospective-specimen collection, retrospective-blinded-evaluation (PRoBE) design. Subjects with incidentally or screening detected IPNs 6–30 mm in diameter with at least 3 consecutive CT scans prior to diagnosis (slice thickness ≤ 1.5 mm) with the same nodule present were included. Disease outcome was adjudicated by biopsy-proven malignancy, biopsy-proven benign disease and absence of growth on at least 2-year imaging follow-up. Lung nodules were analyzed using the Optellum LCP CNN model. Investigators performing image analysis were blinded to all clinical data. The LCP CNN score was determined for 48 benign and 32 malignant nodules. There was no significant difference in the initial LCP CNN score between benign and malignant nodules. Overall, the LCP CNN scores of benign nodules remained relatively stable over time while that of malignant nodules continued to increase over time. The difference in these two trends was statistically significant. We also developed a joint model that incorporates longitudinal LCP CNN scores to predict future probability of cancer. Malignant and benign nodules appear to have distinctive trends in LCP CNN score over time. This suggests that longitudinal modeling may improve radiomic prediction of lung cancer over current models. Additional studies are needed to validate these early findings.

## Introduction

Lung cancer is the leading cause of cancer related death in the United States with a 5-year survival of approximately 22%. Early detection represents the best opportunity for cure as mortality increases with advanced stage^1^. Approximately 1.6 million IPNs are detected annually in the United States alone, the majority of which are benign^2^. Invasive diagnostic procedures on benign nodules result in unnecessary morbidity, mortality, and healthcare costs. A Medicare claims analysis suggests that over 40% of the cost of lung cancer treatment is attributable to the management of adverse outcomes from invasive diagnostic procedures in patients with benign disease^3^. Thus, improving noninvasive methods for diagnosis of IPNs is a focus of extensive research^4^.

Recently, a Lung Cancer Prediction Convolutional Neural Network (LCP CNN) model was trained using the National Lung Screening Trial (NSLT) CT imaging dataset and validated in several external cohorts^5^. The LCP CNN estimates the probability of malignancy for the individual primary nodule with high accuracy and reproducibility and outperforms standard clinical models^5^. The LCP CNN score has also been shown to improve expert CT readers’ ability to correctly classify IPNs as benign or malignant nodules^6^. However, the LCP CNN was trained and validated on single timepoint images with no longitudinal information incorporated into the model (a risk based upon a “snapshot”). Clinically, however, lower risk IPNs are followed over time with serial CT imaging and temporal changes in size or morphology inform management. Similarly, many patients present to clinic with one or more prior CTs already available. Reviewing this prior imaging is critical when deciding on the next diagnostic step^7–9^, yet most radiomic and clinical prediction models do not integrate that information.

Given the improvement in risk stratification of IPNs seen with the LCP CNN based on snapshot assessments, we hypothesized in this pilot study that benign and malignant nodules have different longitudinal trends in LCP CNN scores over multiple timepoints. These trends may reflect different clinical trajectories and improve risk stratification.

## Methods

### Study design

This is a pilot digital biomarker study using a convenience sample. We used a prospective-specimen collection, retrospective-blinded-evaluation PRoBE design^10^. Our primary objective was to evaluate whether the change in LCP CNN scores over time was different between benign and malignant nodules. The LCP CNN score was obtained from prospectively collected CT scans. This study was approved by the Internal Review Boards at Vanderbilt University Medical Center (IRB# 030763, 000616) and the Nashville VA Medical Center (IRB# 310310, 310233). Informed consent was obtained from all patients prior to collection of any data. Research was conducted in accordance with the Declaration of Helsinki.

### Patient selection

Patients were enrolled at Vanderbilt University Medical Center and the Tennessee Valley VA Healthcare System Nashville Campus between 2010 and 2018. Subjects had an initial IPN 6–30 mm in largest axial diameter that was either incidentally or screening detected. Subjects were between 18 and 80 years old at the time of consent and had prospectively collected non-contrast CT scans of the chest with slice thickness ≤ 1.5 mm at the time of initial nodule detection. Subjects had at least 3 sequential CT scans with the same nodule present, with some patients having up to 6 CT scans. If a subject had prior scans with the nodule of interest present, we included those with the reference nodule up 4 mm in size. Disease outcome was adjudicated by biopsy-proven malignancy, biopsy-proven benign, and no evidence of growth on at least 2-year longitudinal imaging follow-up for benign nodules.

### LCP-CNN Score

CT scans were analyzed using the LCP CNN artificial intelligence software developed by Optellum (Optellum LTD, Oxford, UK) as previously described. The LCP CNN is a deep learning model trained in the NLST dataset and externally validated in multiple cohorts^5^. The LCP CNN analyzes raw imaging data from CT scans uploaded to the software and provides an estimate of the probability of malignancy from 0 to 100. The two investigators performing image analysis were blinded to all clinical and image metadata except for the lobe location of the nodule of interest, with no information about the nature of the nodule.

### Statistical analysis

Descriptive statistics are reported as means and standard deviations or median and interquartile range (IQR) for continuous variables and percentages and frequencies for categorical parameters. Comparisons of baseline characteristics between benign and malignant groups were made using Wilcoxon rank-sum for continuous variables and Fisher’s exact test or Chi-squared tests for categorical variables, respectively.

To evaluate the difference in LCP CNN score over time between benign and malignant nodules, a linear mixed effect model was fitted to account for the correlation among LCP CNN score measurements in each patient (i.e., the random intercept effect) and to allow the different change rate for each patient (i.e., the random slope effect). An interaction term between time (months) and groups (benign and malignant nodules) was also included in the model. For each group, the marginal means of linear trends was estimated and was tested with the Wald test. In addition, the difference in trends between groups was evaluated. To further assess the association between LCP CNN scores and the risk of malignancy over time, a joint model for longitudinal and time-to-event data was developed^11^. The joint model was used to predict the conditional probability of non-malignant at time *t*+$\Delta t$ months given event-free survival and the history of LCP CNN scores up to time *t* months for a patient, where $\Delta t$ is the window of prediction. In this model, the mixed-effects models for the longitudinal LCP CNN scores and the Cox proportional hazards model for the time to cancer diagnosis were simultaneously analyzed. The Cox model with piecewise-constant baseline hazard was used for the numerical integration algorithm. The time-dependent area under the receiver operating characteristic (AUC) curves was reported to assess the discriminative capability of a joint model. The LCP CNN score was analyzed on the square root scale to meet the normality assumption. Statistical analysis was performed in R version 4.1.2

## Results

The LCP CNN score was calculated for 80 patients, 48 benign and 32 malignant nodules. The baseline characteristics of the patient cohort are provided in Table 1. The median age of participants was 66 years old (IQR 62–71). Subjects diagnosed with malignancy were older than those diagnosed with benign nodules (64 vs. 69 years old, *p* = 0.03). Most subjects were either smoker or former smokers and there was no statistically significant difference among those diagnosed with benign versus malignant nodules. The median lesion size on the initial CT scan was 10 mm (IQR 7.50–15.25) and there was no difference between the two groups. Most nodules were solid (n = 57, 71.2%) and located in the upper lobes (n = 44, 55%). The most common malignant histologic subtype was adenocarcinoma, which accounted for 75% of cancer diagnoses. The median LCP CNN score for benign nodules was lower than that of malignant nodules 16.75 (IQR 1.57–65.34) versus 35.85 (IQR 14.44–61-42).

**Table 1:** Baseline characteristics of study participants.

	Overall (N = 80)	Benign (N = 48)	Cancer (N = 32)	*p* value
Age, median (IQR)	66 (62–71)	64 (61–70)	69 (65–72)	0.03
Sex				0.002
Female, n (%)	33 (41.2)	13 (27.1)	20 (62.5)	
Male, n (%)	47 (58.8)	35 (72.9)	12 (37.5)	
Smoking history				0.19
Current smoker, n (%)	34 (42.5)	24 (50.0)	10 (31.2)	
Ex-smoker, n (%)	38 (47.5)	19 (39.6)	19 (59.4)	
Never smoked, n (%)	8 (10.0)	5 (10.4)	3 (9.4)	
History of cancer	24 (30.0)	14 (29.2)	10 (31.2)	0.84
Nodule size, median (IQR)	10 (7.50–15.25)	10 (8.6–15.25)	9.30 (6.75–15.25)	0.31
Nodule density				0.36
Part-Solid, n (%)	23 (28.7)	12 (25.0)	11 (34.4)	
Solid, n (%)	57 (71.2)	36 (75.0)	21 (65.6)	
Spiculation, n (%)	26 (32.5)	11 (22.9)	15 (46.9)	0.03
Location				0.3
LLL, n (%)	14 (17.5)	11 (22.9)	3 (9.4)	
LUL, n (%)	20 (25.0)	9 (18.8)	11 (34.4)	
RLL, n (%)	14 (17.5)	10 (20.8)	4 (12.5)	
RML, n (%)	8 (10.0)	5 (10.4)	3 (9.4)	
RUL, n (%)	24 (30.0)	13 (27.1)	11 (34.4)	
Histology				
Adenocarcinoma, n (%)			24 (75.0)	
Squamous Cell Carcinoma, n (%)			4 (12.5)	
Carcinoid, n (%)			2 (6.2)	
Large cell carcinoma, n (%)			1 (3.1)	
Small Cell Carcinoma, n (%)			1 (3.1)	
LCP CNN score, median (IQR)	25.78 (2.65–65.34)	16.75 (1.57–65.34)	35.85 (14.44–61.42)	0.17

*IQR* Interquartile range; *LLL* Left lower lobe; *LUL* Left upper lobe; *RLL* Right lower lobe; *RML* Right middle lobe; *RUL* Right upper lobe.

**Figure 1:**
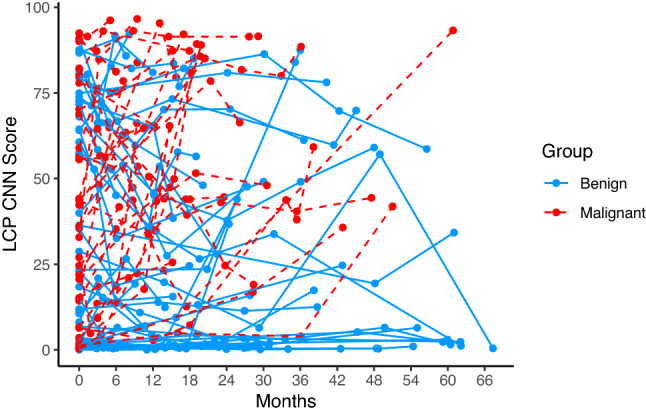
Spaghetti plot with LCP CNN score over time for benign and malignant nodules.

**Figure 2:**
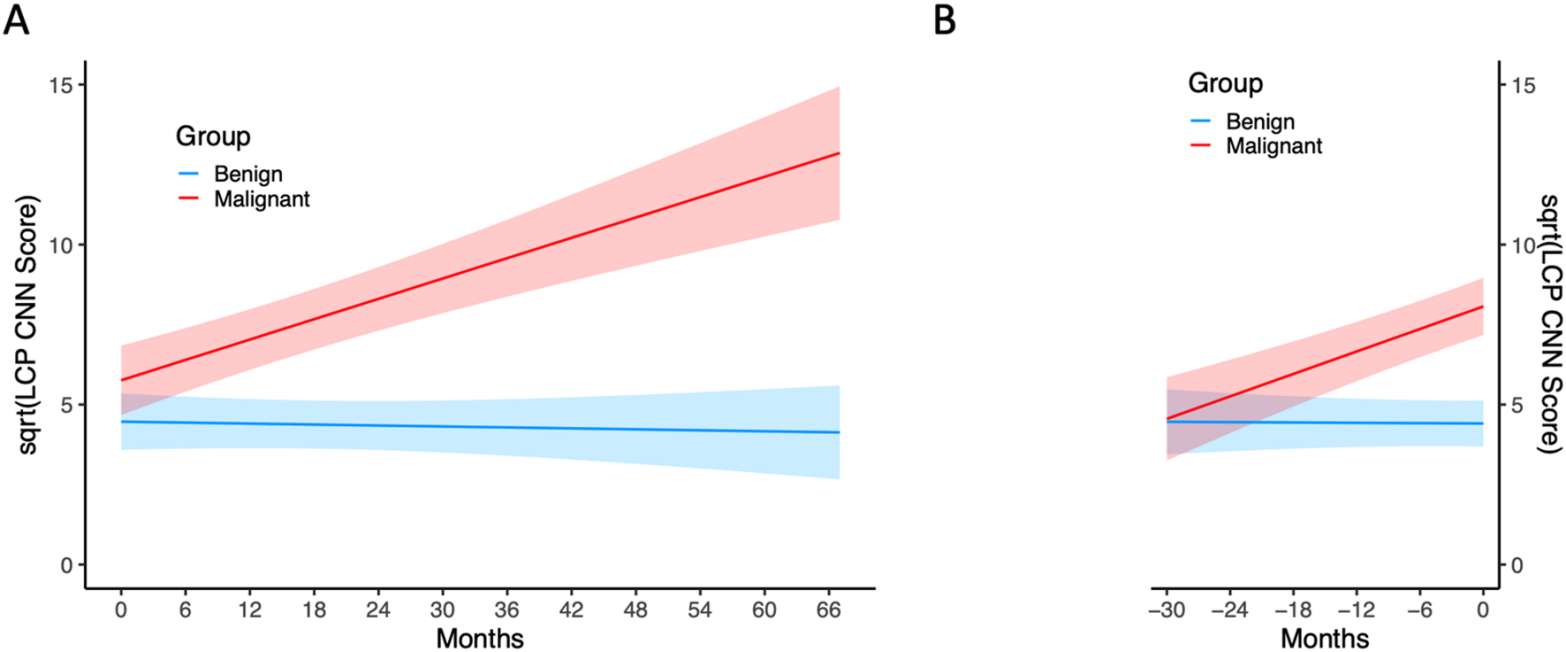
The predicted LCP CNN score on a square root scale overtime for benign and malignant nodules. Panel A shows the predicted LCP CNN score with time 0 representing the time of initial nodule identification. Panel B shows the predicted LCP CNN score with time 0 representing the time of final nodule diagnosis.

Raw data of LCP CNN scores over time is presented in Fig. 1. Subjects with benign nodules are shown in blue, and subjects with cancer are shown in red. The LCP CNN scores were variable over time. In general, they tended to increase over time for malignant nodules while benign nodule scores tended to remain stable. A mixed effect model was then used to analyze the LCP CNN score over time on the square root scale to meet the normality assumption. There was a significant difference in the probability trend between benign and malignant nodules (the difference in trend = 0.111, *p* < 0.001), Table 2. Overall, the LCP CNN score of benign nodules remained relatively stable over time while that of malignant nodules continued to increase over time (trend = 0.106 for malignant patients, *p* < 0.001; trend = − 0.005 for benign patients, *p* = 0.669), Table2 and Fig. 2.

**Table 2:** Trend estimation and difference in trend of LCP CNN scores overtime (on a square root scale).

Hypothesis	Estimate	Standard error	*p* value
Trend_malignant_ = 0	0.106	0.018	< 0.001
Trend_benign_ = 0	− 0.005	0.013	0.699
Trend_malignant_ − Trend_benign_ = 0	0.111	0.022	< 0.001

**Figure 3:**
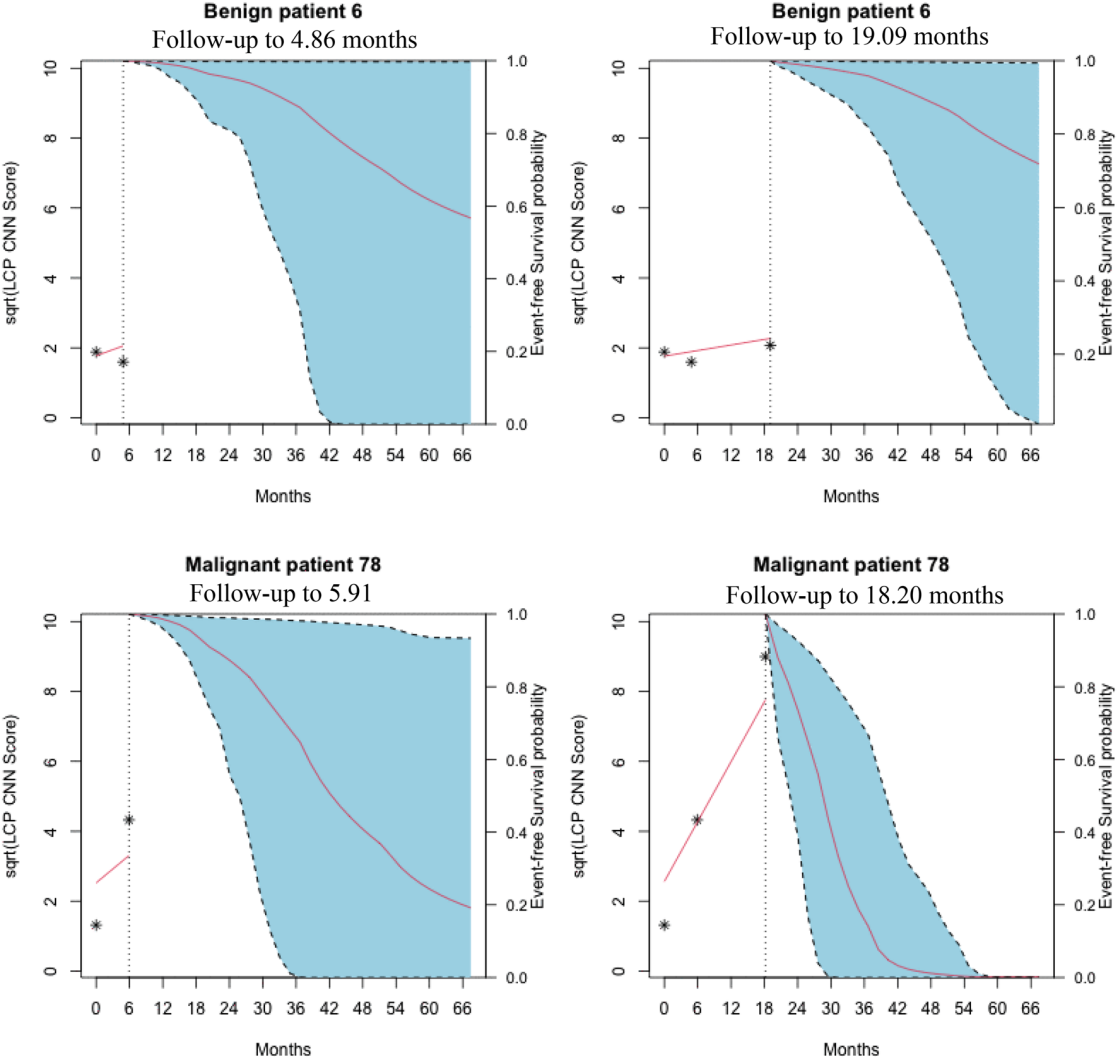
Dynamic predicted probabilities of event-free (non-malignant) survival for a benign patient 6 (top panel) and a malignant patient 78 (bottom panel) estimated with the joint model. The LCP CNN longitudinal score (star points) for the second visit (left panel) and the third visit (right panel) are shown. The vertical dotted lines represent the time point of the last probability. Left of the vertical line, the fitted longitudinal trajectory (solid line) is depicted. Right of the vertical line, prediction of the conditional probabilities of event-free survival (solid line) with 95% confidence intervals (dashed line).

**Figure 4:**
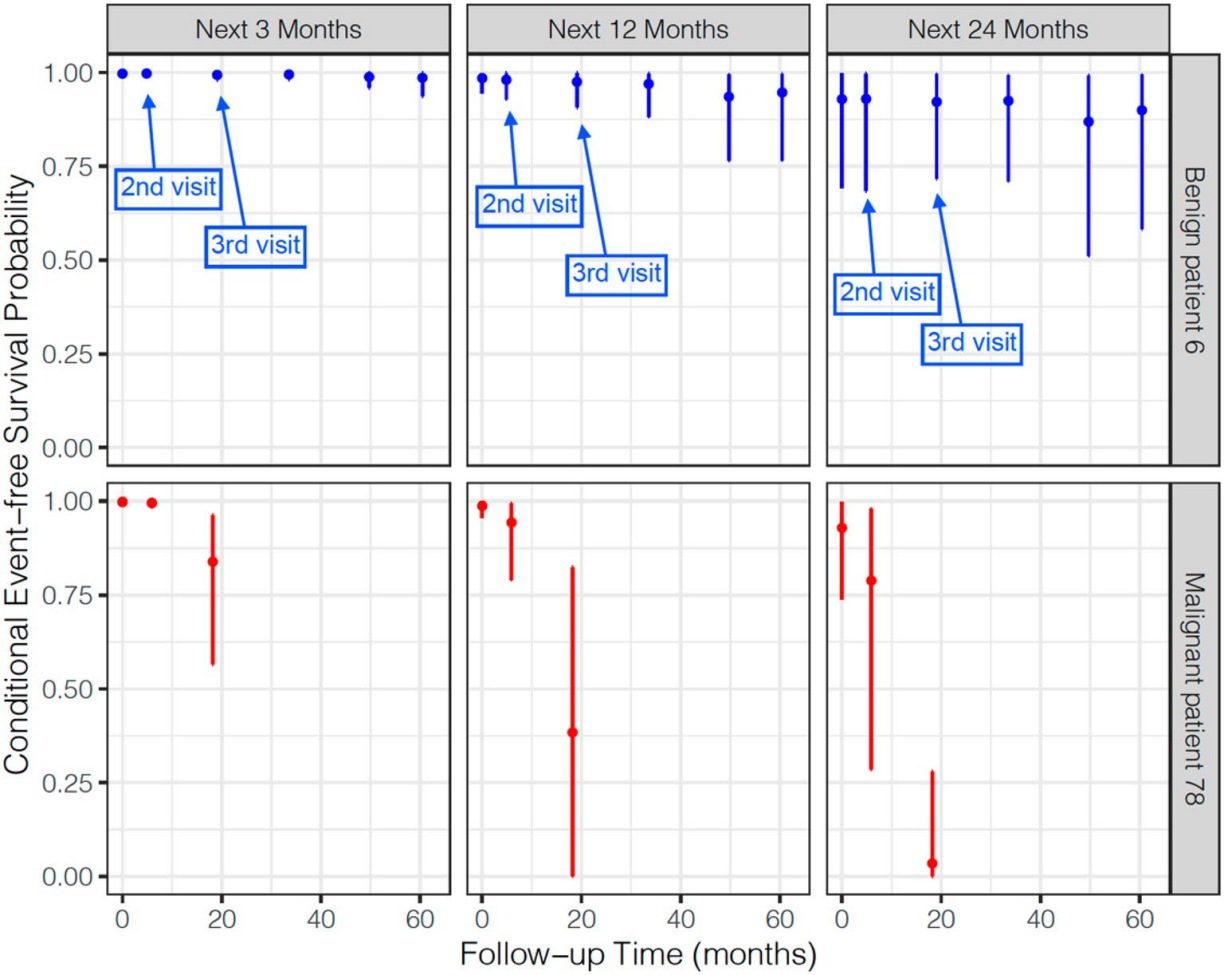
Conditional event-free survival (probability) with 95% confidence intervals for the benign patient 6 (top panels) and the malignant patient 78 (bottom panels) at t + 3 (left panels), t + 12 (middle panels), and t + 24 months (right panels), where t represents follow up visits. In this example, benign patient 6 had six visits and malignant patient 78 had three visits.

To further evaluate the association between prior LCP CNN scores and the probability of cancer over time, we fit a joint model, which predicts the conditional probability of event-free survival (non-malignant) at a future time point (*t* + $\Delta t$) given the longitudinal LCP CNN score up to time (t) for each subject. The dynamic prediction probabilities of event-free survival for two subjects, 6 and 78 are presented in Figs. 3 and 4. The dynamic survival probabilities at *t*+$\Delta t$ in Fig. 4 are selected at $\Delta t$=3, 12, and 24 months. For the benign subject (top panel of Figs. 3 and 4), given the LCP CNN scores up to t = 4.86 months (the second visit), the probabilities of event-free survival with 95% confidence interval were 0.9975 (0.9901, 0.9998) at next 3 months, 0.9808 (0.9291, 0.9989) at next 12 months, and 0.9297 (0.6848,0.9970) at next 24 months. After dynamic scores were updated up to t = 19.09 months (third visit), the survival probabilities at next 3, 12 and 24 months slightly decreased as shown in Fig. 4. The bottom panels of Figs. 3 and 4 illustrates the probabilities of event-free survival with 95% confidence intervals for the cancer subject. As shown, as the LCP CNN score increased over time the event free survival decreased.

**Figure 5:**
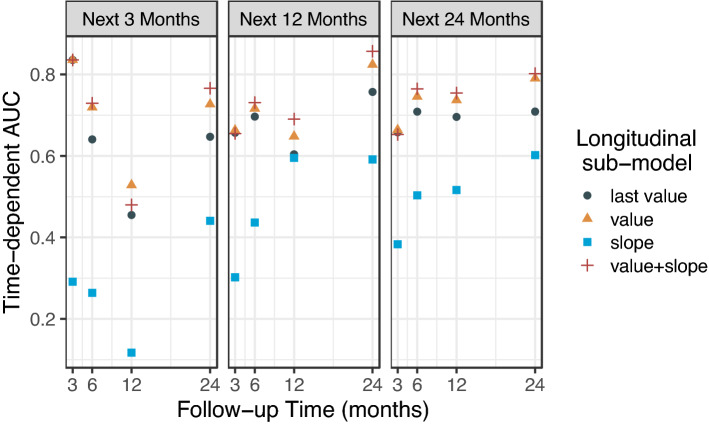
Time-dependent AUC under four different longitudinal sub-models in the joint model for time interval (*t*, *t* + 3] (next 3 months, left panel), (*t*, *t* + 12] (next 12 months, middle panel), and (*t*, *t* + 24] (next 24 months, right panel) given the longitudinal LCP CNN scores to *t* = 3, 6, 12, and 24 months. The sub-model that includes the longitudinal LCP CNN scores plus the change rate performed the best among the four sub-models. Last value—last LCP CNN score, value—longitudinal LCP CNN score, slope—rate of change in LCP CNN score, value + slope—longitudinal LCP CNN score plus rate of change in LCP CNN score.

The time-dependent AUCs were then used to evaluate the discrimination of the joint model. For LCP CNN scores collected up to time t = 3, 6, 12, 24 months, the time-dependent AUCs within next 3 months (i.e., (*t*, *t* + 3]) next 12 months and next 24 months were estimated. We hypothesized that the joint-model with the longitudinal LCP CNN scores performed better than a cross sectional logistic regression model with a single time point. The time-dependent AUC curves were used to quantify the discriminative ability of LCP CNN scores at each time point under consideration for the censored event (malignancy) times. However, logistic regression was evaluated using the classic approach of ROC curve analysis and ignoring the time dependency of the disease status^12^. Therefore, to be comparable with the joint-model, we included the last score only in the longitudinal sub-model in the joint-model representing a special case of logistic regression. We were also interested in the effect of score change over time (slope) on discrimination. We considered several longitudinal sub-models including last score only (i.e., single point for each patient, so the random slope effect was not applied), the slope term of the score, as well as the value plus slope term. As shown in Fig. 5, the sub-model that includes the longitudinal scores and change rate (value + slope) outperformed the other sub-models. The sub-model with the longitudinal scores (value) had a higher time-dependent AUC than the one with a single score (last value). The sub-model with change of rate (slope) over time performed the worst among the four models. Note that the time-dependent AUC varies with the longitudinal follow-up time t, the window of prediction $\Delta t$, and number of malignant diagnoses within time interval (t, t + $\Delta t]$.

## Discussion

Most quantitative methods for estimating the pretest probability of malignancy for IPNs incorporate a snapshot of data at a single timepoint. However, in clinical practice, nodule follow up and analysis of growth or morphological change over time is an extremely important step in the management algorithm^7–9^. In this pilot biomarker study, we have shown that the LCP CNN score trends differ across benign and malignant nodules. Overall, benign nodules have relatively stable LCP CNN scores while malignant nodules tend to have continuously rising scores. This makes intuitive sense, as clinically benign nodules remain stable or even regress over time while malignant nodules tend to grow and acquire morphological features associated with malignancy. Nonetheless, this distinction is not universal as in some instances, as illustrated in this study, some benign nodules exhibit growth patterns over time that mimic those of their malignant counterparts. In these cases, additional diagnostic evaluation in the form of biopsy or positron emission tomography (PET) is often pursued. Longitudinal LCP CNN scores may be useful in these cases, as relatively stable scores could provide some reassurance in the right clinical context and prevent unnecessary investigations, as suggested in this study. Similarly, malignant nodules could have a rising score in the absence of appreciable changes on CT imaging and raise concerns. A rising LCP CNN score in this context might be an indication that the nodule is malignant despite this relative stability which would allow for an earlier intervention.

For IPNs with low-intermediate probability of cancer, being able to predict the future probability of cancer with high accuracy is very appealing. This could allow the provider to change their management from PET or biopsy to surveillance, alter the time interval for CT follow up (e.g. from 6 to 12 months), thus reducing unnecessary radiation exposure, morbidity, mortality and healthcare costs. In this study, we used a joint model to estimate the future probability of cancer for a given follow up time based on the longitudinal LCP CNN scores. Our results suggest that the discriminative capability of the joint model with longitudinal data (either the sub-model with longitudinal scores or the sub-model with scores plus slope) performs better than the sub-model that includes only the last LCP CNN score. For example, after LCP CNN scores are collected up to 24 months, the time-dependent AUC within the next 12 months is 0.757 for the sub-model that includes only the last LCP CNN score versus 0.857 for the sub-model that includes the longitudinal scores plus their slope. While promising, these early findings need to be validated.

Changes in radiomic features over time (“delta radiomics”) is a promising but underexplored area of research^13^. Some studies have shown that changes in radiomic features over time might be better at predicting lung cancer in the context of lung cancer screening^14, 15^. In the field of lung cancer, most of the studies on delta radiomics have used conventional radiomics, which currently requires nodule segmentation and expert review, and have focused mainly on predicting response to therapy, survival, mutational status, and lung cancer screening. Longitudinal trends have also been explored using deep learning models, but so far, these analyses have been limited to the screening setting^16^. The LCP CNN score uses a commercially available deep learning algorithm that does not require nodule segmentation or expert input, so that if confirmed, these preliminary data would be immediately clinically relevant. To our knowledge, this is the first study to evaluate longitudinal trends on both, incidental and screening detected nodules, using a commercially available and FDA cleared radiomic algorithm.

This study has several limitations including the convenient sample used, the limited sample size, and the lack of a validation dataset. The study population is composed of patients with multiple CT scans available for analysis within the research biorepository and is therefore not necessarily representative of a particular clinical population. Additionally, as routinely happens in clinical practice, the intervals between scans were highly variable and many subjects did not have scans at 3 and 6 months. Hence, the model discriminatory ability is affected at these early timepoints. Similarly, many subjects do not have more than 2 years follow up and this could also affect the model’s performance. These shortcomings could be overcome in future studies by enrolling subjects with shorter and longer interval follow ups. Another potential limitation and source of error is the use of different CT scanners and acquisition protocols. However, we only included non-contrast CT scans with slice thickness ≤ 1.5 mm. Furthermore, the LCP CNN model was trained in the NLST dataset and validated in multiple cohorts from 2 different countries^5^, which would represent numerous CT scanners and protocols. We do not have the radiologist assessment or recommendation for many of the CT scans as they were from outside institutions and radiology reports were not available. It is possible that the radiologist assessment of the nodule could be as good or better than the LCP CNN model, although a recent study showed that the LCP CNN score improves readers’ ability to correctly classify pulmonary nodules as benign or malignant^6^. Because the analysis was based on a limited retrospective dataset, we can only infer from a separation of the probability score trajectories that occurs before the time of diagnosis that such longitudinal analysis would in reality lead to earlier diagnosis, treatment and improved patient outcomes. It is possible that these trends are introduced by biases inherent in the retrospective design of our study since patients present to lung nodule experts at widely different timepoints in the course of their disease. Future work based on prospective evaluation of these trends will be needed to confirm our hypothesis.

Despite these limitations, this pilot digital biomarker study suggests that malignant and benign nodules have different LCP CNN score trends over time, which is promising. We also developed a joint model that incorporates longitudinal LCP CNN scores to predict future probability of cancer which if validated, could be readily implemented to improve the clinical utility of a commercially available radiomic biomarker. A larger study is underway to ensure model calibration, to compare to size trend over time, and to validate in an external cohort (5R01CA253923).

## Data Availability

The datasets used and/or analyzed during the current study available from the corresponding author on reasonable request.
